# Advances in nutritional interventions for coronary heart disease patients from the perspective of the gut-heart axis

**DOI:** 10.3389/fnut.2025.1676619

**Published:** 2025-11-14

**Authors:** Jing Gao, Mingjing Zhang, Gaoning Zhang, Dingzheng Zhang, Mo Zhou, Lijing Zhao, Yanwei Du

**Affiliations:** 1 School of Nursing, Jilin University, Changchun, Jilin, China; 2 Department of Nursing, First Affiliated Hospital of Xinjiang Medical University, Urumqi, Xinjiang, China; 3 Institute of Orthopaedic & Musculoskeletal Science, Royal National Orthopaedic Hospital, University College London, London, United Kingdom

**Keywords:** gut-heart axis, coronary heart disease, nursing, nutrition, gut microbiota

## Abstract

Coronary heart disease (CHD) is the most common type of cardiovascular disease (CVD) and poses a heavy economic burden worldwide due to its persistently high incidence and mortality rates. In recent years, the pathogenesis of CHD is well-understood, and the “gut-heart axis” theory reveals that the gut microbiota mediated dietary components enter the cardiovascular system via mechanisms including immune metabolism, epigenetics (regulation), and microbial metabolism, which can promote the formation and development of atherosclerosis. This theory identifies the gut microbiota as a promising therapeutic target for CHD intervention. The gut microbiota is highly plastic and closely related to dietary habits. Appropriate nutritional management can achieve the purpose of preventing and treating CHD. From the perspective of the “gut-heart axis,” this review integrates previous research results and current research progress on the gut microbiota in CHD, summarizes the gut microbiota and related pathogenesis in CHD patients, discusses the interrelationship between CHD, nutritional management, and gut microbiota, and explores the existing problems and recent advances in nutritional management of CHD patients, which provides reference ideas for possible therapeutic strategies and precision nutrition support in CHD.

## 1 Introduction

Coronary heart disease (CHD) is the most common type of cardiovascular disease (CVDs). It is caused by chronic inflammation leading to the thickening of the arterial walls, thus leading vascular narrowing or obstruction and the formation of atheromatous plaques, which can further result in myocardial ischemia, hypoxia, or even necrosis, causing functional impairments and organic lesions of the heart (1). According to data released by the World Health Organization (WHO) in 2021, approximately 18.6 million people died from cardiovascular diseases, representing 31% of the total global mortality and ranking as the leading cause of death worldwide (2). Notably, CHD accounts for nearly 50% of these deaths. Although advances have been made in CHD prevention and treatment (3), the incidence and mortality rates of CHD still remain high globally (4), associated medical expenditures impose a huge economic burden on the world.

The gut microbiota is a complex and dynamic ecosystem of microorganisms residing in the human intestinal tract. These microorganisms play important physiological functions in promoting glucose metabolism, protein hydrolysis, stimulation of the immune system, enhancement of innate immunity against pathogens, and modulation of the mucosal barrier (5). Dysbiosis of the gut microbiota can lead to an enhanced inflammatory response in the body, disrupting intestinal barrier function, allowing endotoxins to enter the circulation and trigger a systemic inflammatory response (6, 7). Chronic inflammation is an important pathological basis of CHD (8, 9). Based on comprehensive genetic data from over 450, 000 individuals of European ancestry, a causal genetic susceptibility linking gut microbiota to CHD has been established. Moreover, the gut microbiota is associated with various risk factors for CHD, including obesity, diabetes, hypercholesterolemia, and hypertension (10). It has even been found that some gut microbiota may be potential risk factors for CHD (11).

The theory of the “gut-heart axis” was first proposed by Stanley Hazen’s team, highlighting the close relationship between the gut microbiota and cardiovascular health (12). The composition of the gut microbiota is strongly influenced by host’s dietary habits (13). As a key component in both the prevention and treatment of CHD, scientific and rational nutritional management is of great significance for improving lipid profiles, controlling body weight, lowering blood pressure, and reducing the risk of cardiovascular events (14, 15). This review focuses on the gut-heart axis and discusses from three perspectives: CHD, gut microbiota, and nutritional management, aiming to provide a novel approach of precise nutritional support for CHD patients.

## 2 Epidemiology and socioeconomic burden of coronary heart disease

In 2022, the global prevalence of CHD reached 315 million cases, with an age-standardized prevalence rate of 3,605 cases per 100,000 population (16, 17). The incidence of coronary heart disease increases significantly with age (18). Among individuals over 65 years old, male CHD patients account for 33%–65% of atherosclerotic cardiovascular events, whereas female patients account for 28%–58%. Males are more likely to develop CHD at a younger age than females; however, the incidence of CHD in females rises rapidly after menopause. According to a report (19), although the global age-standardized mortality rate of CVDs declined by 34.9% from 1990 to 2022, the absolute number of CVD-related deaths increased from 12.4 million in 1990 to 19.8 million in 2022, reflecting the shifts in global population structure and the growing burden of chronic diseases. The World Health Organization (WHO) estimates that by 2030, the global population aged 60 years and older will increase by 34%, rising from 1 billion in 2019 to 1.4 billion; by 2050, the global elderly population is expected to exceed 2.1 billion (20). With the aging of the population, the prevalence and mortality of cardiovascular diseases will inevitably increase rapidly.

The epidemiological status of CHD varies significantly across different regions. In developed countries, the incidence and mortality rates have declined and leveled off due to improved medical conditions and control of risk factors. In the United States, the prevention and management of CHD presents a complex situation of “overall improvement with local deterioration.” The incidence and mortality rates of CHD have declined significantly since 1990 (21), although it remains one of the leading causes of death (22). According to the American Heart Association’s Global Cardiovascular Disease Statistics report, approximately 19.4 million deaths occur annually due to CVDs, an increase of 68% from 1991, of which ischemic heart disease causes nearly 9 million deaths (46.39%) and 129 million disabilities (23). Among U.S. states, Mississippi bears the highest burden of metabolic risk factors, while Arkansas stands out for its high rates of hypercholesterolemia and smoking. In northeastern cities such as Boston and Baltimore, the prevalence of CHD is significantly elevated (21, 24). From 2005 to 2019, the CVDs mortality rate in Europe showed a declining trend, with the rate for female decreasing from 1.752 to 1.662 per 100,000 population; and the rate for male decreasing from 3.372 to 3.135 per 100,000 population, but there is still a significant gap between the east and west (25, 26). Western European countries such as France, the Netherlands, and the United Kingdom have experienced a significant decline in CHD mortality rates (over 60%) since 1980’s. European countries like France and Spain have become one of the lowest CHD mortality rates in Europe (27–29). In contrast, Eastern European countries such as Hungary, Romania, Croatia, and Lithuania have shown less improvement (30, 31). These disparities may be attributed to differences in the healthcare resource allocation, the implementation of preventive policies, and socioeconomic factors (32). The prevalence of CHD in Africa is showing a rapidly increasing trend, with a significantly higher prevalence among males compared to females (33), and onset occurring at a younger age than that of European and American populations. Between 1990 and 2019, the age-standardized mortality rate of coronary heart disease in Sub-Saharan Africa (SSA) declined by 14.4%, but the crude number of cases increased by 131.7% (34, 35). The Central African Republic, Madagascar, and Lesotho bear the highest CHD burden in the region (36). In South Africa, the self-reported prevalence of CHD is 1.29%, which is much lower than that of stroke (4.29%) (37). However, underdiagnosis in parts of West and Central Africa may lead to underestimation of the actual CHD prevalence (36). In 2021, the number of cardiovascular disease cases in Southeast Asian countries was 36.8 million, and the number of deaths from cardiovascular disease was 1.66 million (ranging from 1.51 to 1.80 million), making it the leading cause of disease burden in the region (2).

According to data from the Journal of the American College of Cardiology: Asia, more than half of global CVD deaths occurred in Asia, and this will continue to rise in the coming decades, with a trend toward younger age of onset (38). Compared with 1990, the number of CVD patients in Asia increased by 148.1% (144.0%–152.5%), while the age-standardized prevalence increased by 2.5% (1.4%–3.6%). The mortality rate of CHD in Japan has long been at a low level globally, with the dual characteristics of “low mortality but rising potential risk.” In 2015, the age-standardized mortality rate was 17 per 100,000 (male), a significant decrease from 1980 (40/100,000), but in recent years, there has been a potential upward trend of the Age-standardized mortality rate (ASMR) in the urban population of young men (39, 40). In India, the CHD mortality rate among female increased by 47.4% from 2000 to 2017, exceeding the growth rate of male (32.8%) (41, 42). The China Cardiovascular Health and Disease Report 2023 (43) estimates that there are 330 million people in China are currently living with cardiovascular disease, with an overall annual mortality rate showing an upward trend, with male higher than female, and the annual prevalence rate in rural areas higher than in urban areas and increasing year by year.

Despite notable progress in the treatment and prevention of CHD, its global incidence and mortality rates remain persistently high. In the United States, the cost of a single coronary artery bypass graft (CABG) operation is approximately US$ 150, 000, with annual CHD-related healthcare expenditures exceeding $220 billion (22). In China, premature deaths caused by CVDs result in a GDP loss of 1.5% (44). Studies have shown that caregivers of CHD patients need to spend an average of more than 25 h per week assisting with daily activities, which places significant pressure on family members (45), and leads to a 22%–28% reduction in employment rates among them (46). According to data from the Global Burden of Disease (GBD) Study (36), the global age-standardize incidence rate of CHD among people aged 1–79 years increased from 177.1 per 100,000 person-years in 1990 to 203.7 in 2010, and then decreased to 197.4 in 2019. Globally, the number of disability-adjusted life years (DALYs) caused by CHD reached 180 million each year, and increase of 33.7%, of which the increase in males was 51.8%, much higher than that in females (12.1%).

## 3 Alterations in the gut microbiota of patients with coronary heart disease

**FIGURE 1:**
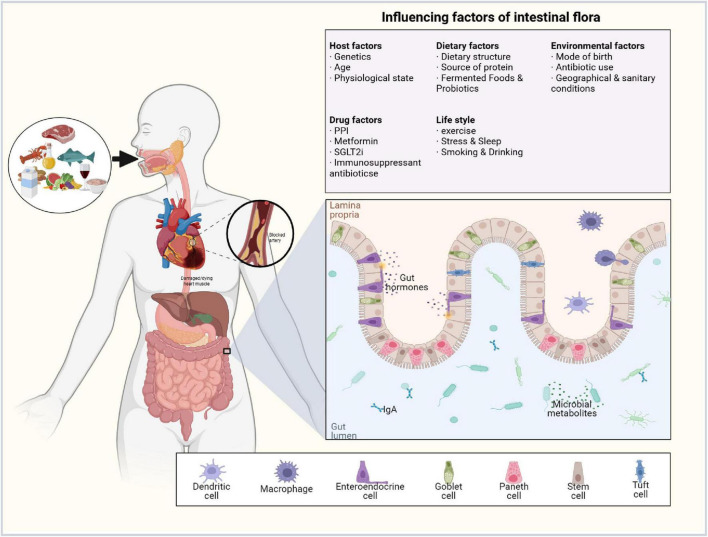
Composition of gut microbiota and its influencing factors. Created in BioRender. Gao, J. (2025) https://BioRender.com/h4f0ctv.

Structural dysbiosis of the gut microbiota in patients with CHD has emerged as a central focus in the study of the gut-heart axis (Figure 1). A synthesis of relevant studies (47–49) yields the following consistent conclusions: (1) A marked reduction in α-diversity—Faith, Chao1, Shannon, and Simpson indices were all lower in the CHD group compared to healthy controls, indicating a concurrent decline in both richness and evenness of the microbiota; (2) Clear separation in β-diversity—PCoA and PERMANOVA analyses revealed a “clustered deviation” in the gut microbial composition of CHD patients, with a significant increase in distance from healthy controls, which positively correlated with the number of affected coronary arteries and Gensini scores; (3) A “two-decrease-two-increase” pattern at the phylum level: decreased relative abundances of *Bacteroidota* and *Actinomycetota*, alongside significant enrichment of *Bacillota* (including butyrate-producing bacteria such as *Lachnospiraceae* and *Ruminococcaceae*) and Proteobacteria (*predominantly Enterobacteriaceae*).

Diminished gut microbial diversity is directly associated with alterations in metabolite profiles: a 30%–45% reduction in butyrate-producing bacteria leads to decreased luminal butyrate concentration, impairing GPR43/109A signaling and downregulating epithelial tight junction protein expression, resulting in increased intestinal barrier permeability and a 2–4-fold elevation in plasma LPS levels. Concurrently, the expansion of facultative anaerobes such as *Enterobacteriaceae* and *Streptococcaceae* enhances choline-TMA lyase (cutC/D) activity, promoting upregulation of hepatic TMAO synthesis pathways and a 1.8–3.2-fold increase in serum TMAO levels, which exhibits a linear positive correlation with LDL-C and systolic blood pressure. Network co-occurrence analysis further reveals that the gut microbiota of CHD patients is characterized by an “opportunistic pathogen cluster” (*Streptococcaceae, Erysipelotrichaceae, Enterococcaceae*) as core nodes, whereas healthy controls exhibit a “SCFA-producing cluster” (*Bacteroidaceae, Lachnospiraceae, Rikenellaceae*) as hubs. These clusters demonstrate negative connectivity, indicating that niche occupation by pathogenic microbiota serves as a key driver of diversity reduction.

Coronary heart disease patients exhibit reduced gut microbiota diversity, which extends beyond mere quantitative depletion to encompass a systemic imbalance characterized by inverted “beneficial-pathogenic” bacterial ratios, metabolic reprogramming, and compromised gut-blood barrier integrity. This dysbiosis accelerates atherosclerosis progression via the “low-grade endotoxemia–TMAO–inflammation” tripartite axis.

Koren et al. identified *Chryseomonas*, *Clostridium*, and *Streptococcus* species in atherosclerotic plaque samples, which were the same strains present in the gut microbiota, and their presence was also associated with cholesterol levels (50), suggesting that gut microbes may directly contribute to the development of atherosclerosis. Studies have shown the specific gut microbiota will also change in patients with CHD, including a significant increase in the abundance of *Collinsella*, and a decrease in *Rothia* and *Eubacterium* species. Additionally, the relative abundance of *Enterobacteriaceae* and *Streptococcus* is higher in CHD patients compared to healthy controls (51), which may be associated with reduced anti-inflammatory capacity. There are also contrary results, such as a significant increase in the abundance of mature *Lactobacillus* species, along with a marked decrease in beneficial bacteria such as *Bifidobacterium* and *Prevotella* in CHD patients (52). These inconsistencies may be attributed to differences in participants’ living environments, dietary habits, sampling procedures, and specimen storage conditions.

Pharmaceutical agents and gut microbiota engage in bidirectional regulatory interactions (53–55). Through dual pathways of bacterial inhibition and promotion, drugs can remodel the gut microbiota structure in patients with coronary heart disease, consequently influencing cardiovascular outcomes. Statins (*rosuvastatin* and *atorvastatin*) elevate butyrate-producing bacteria such as *Bifidobacterium* and *Anaerostipes*, while suppressing pro-inflammatory bacteria like *P. merdae*. They concurrently increase secondary bile acids and short-chain fatty acids (SCFAs), enhance cholesterol efflux, and serve as predictive biomarkers for therapeutic efficacy. Low-dose aspirin increases *Bacteroidetes*, reduces the Firmicutes/Bacteroidetes (F/B) ratio, restores tight junction proteins, and decreases LPS entry into the bloodstream. It synergizes with the enrichment of *Parabacteroides goldsteinii* to exert anti-atherosclerotic effects. In contrast, high-dose aspirin may increase intestinal permeability, necessitating careful dose consideration.

In the realm of glucose-lowering medications, metformin, GLP-1 receptor agonists (GLP-1RAs), and SGLT2 inhibitors have been shown to promote the growth of short-chain fatty acid (SCFA)-producing bacteria such as Roseburia and Faecalibacterium, while suppressing trimethylamine N-oxide (TMAO)-producing bacteria like Escherichia-Shigella. These mechanisms contribute indirectly to improved insulin sensitivity and reduced cardiovascular events. The ACE inhibitor captopril enhances intestinal mucosal structure, reduces permeability, and reverses hypertension-associated gut microbiota dysbiosis. Rifaximin exerts a “pro-symbiotic” effect in patients with heart failure and intestinal edema by selectively inhibiting aerobic Gram-negative bacteria, reducing bacterial translocation, and restoring microbial alpha-diversity.

The efficacy of digoxin is influenced by its metabolism through *Eggerthella lenta*, with approximately 10% of patients experiencing loss of therapeutic effect due to microbial community variations.

The emerging “drug-the-bug” strategy employs the TMA inhibitor 3,3-dimethyl-1-butanol to target microbial CutC/D enzymes, significantly reducing trimethylamine N-oxide (TMAO) levels and reversing atherosclerosis. Concurrently, the gut microbiota metabolite phenylacetylglutamine (PAGln) promotes platelet aggregation via β2-adrenergic receptor activation, positioning it as a potential novel therapeutic target. Drug–microbiota interactions exhibit bidirectional regulation, and integrating microbiota profiling to guide personalized medication holds promise for enhancing the efficacy of secondary prevention in coronary heart disease while reducing adverse drug reactions.

The distribution and composition of the gut-associated mesenteric microbiota are influenced by various factors, including genetic elements (e.g., HLA-DRB, alleles), geographical conditions (e.g., altitude and region), age, and sex. These factors reflect that there are obvious differences between the mesenteric microbiota of patients with CHD and that of healthy individuals. Since gut microbial dysbiosis can lead to disturbances in energy metabolism, resulting in obesity, insulin resistance, and other metabolic disorders, and can directly involved in the process of blood pressure and blood glucose regulation, thereby certain impacting cardiovascular function. A range of gut microbiota derived metabolites have been associated with CHD risk, including trimethylamine N-oxide (TMAO), short- chain fatty acids (SCFAs), secondary bile acids (e.g., deoxycholic acid (DCA), lithochoic acid (LCA), phenylacetylglutamine (PAGln), lipopolysaccharide (LPS). These metabolites hold potential as predictive biomarkers of CHD and as valuable references for personalized treatment. For CHD patients, nutritional interventions strategies that target the modulation of gut microbiota may represent a novel therapeutic target for cardiovascular disease (56–60).

## 4 Exploration of the mechanistic pathways of the gut–heart axis theory

### 4.1 Production of pro-atherogenic metabolites

Dietary nutrients, such as choline and L-carnitine, are metabolized by specific gut microbiota, such as *Clostridium spp* and *Lactobacillus spp*, to produce trimethylamine (TMA), which is subsequently oxidized in the liver by flavin-containing monooxygenase 3 (FMO_3_) to form trimethylamine N-oxide (TMAO). TMAO inhibits the farnesoid X receptor (FXR) signaling pathway, leading to reduced expression of the cholesterol efflux transporter ABCA1, thereby promoting macrophage foam cell formation and expansion of the lipid core of atherosclerotic plaques (61). Meanwhile, TMAO activates the NLRP3 inflammasome and the platelet TLR4/ERK5 pathway, inducing IL-1β release and further enlarging the plaque necrotic core (62), and it also enhances platelet P2Y12 receptor signaling, thereby promoting thrombosis (63). Additionally, tryptophan and tyrosine are metabolized by gut microbes, such as *Bacteroides spp*, leading to activation of the aryl hydrocarbon receptor (AhR), which induces reactive oxygen species (ROS) accumulation in endothelial cells, resulting in mitochondrial dysfunction and endothelial senescence (64, 65).

### 4.2 Loss of protective metabolites

**FIGURE 2:**
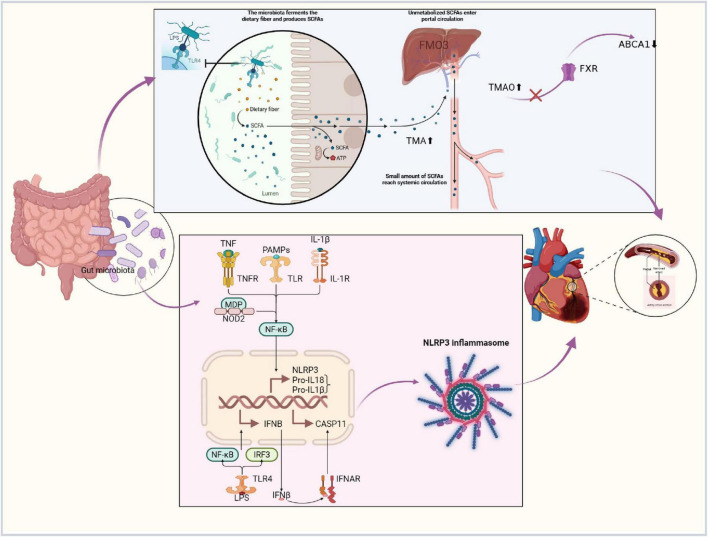
Exploration of the mechanistic pathways of the gut–heart axis theory. Created in BioRender. Gao, J. (2025) https://BioRender.com/h4f0ctv.

Gut microbiota produces short-chain fatty acids (SCFAs), such as acetate, propionate, and butyrate by fermenting dietary fiber, which play essential roles in host energy metabolism and immune regulation. Dysbiosis of the gut microbiota leads to a reduction in metabolites with cardiovascular-protective effects, thereby exacerbating the progression of atherosclerosis. Deficiency of SCFAs, for instance, butyrate, compromises the integrity of the intestinal barrier, facilitating the translocation of lipopolysaccharides (LPS) into the bloodstream. This process activates the systemic TLR4/NF-κB inflammatory cascade and upregulates the expression of endothelial adhesion molecules (ICAM-1 and VCAM-1), promoting monocyte infiltration and vascular inflammation (66). Secondary bile acids, such as deoxycholic acid (DCA), reduce plaque repair ability by activating TGR5 receptors on vascular smooth muscle cells, suppressing M2 macrophage polarization, inhibiting autophagy, and promoting the formation of calcified nodules (67). In addition, Gut microbiota dysbiosis can regulate the Th17/Treg immune balance through tryptophan metabolites (such as kynurenine) (68, 69), thereby influencing the local inflammatory microenvironment of atherosclerotic plaques. Based on the “oral-gut-vascular axis,” the gut microbiota and oral microbiota, such as *Porphyromonas gingivalis*, can synergistically activate the TLR2/MMP-9 signaling pathway, accelerating the degradation of the fibrous cap of plaques and consequently exacerbating plaque instability (70) (Figure 2).

### 4.3 Dysregulation of the immune–inflammatory network

(1)    Innate immune activation: Increased intestinal permeability leads to translocation of lipopolysaccharide (LPS) into the bloodstream, activating Toll-like receptor 4 (TLR4) on endothelial cells and promoting the release of pro-inflammatory cytokines such as IL-6 and TNF-α, thus inducing systemic ow-grade inflammation. Trimethylamine N-oxide (TMAO) stimulates platelet release of high-mobility group box 1 (HMGB1), triggering neutrophil extracellular trap formation (NETosis), which contributes to plaque erosion (71, 72) (Figure 2).
(2)    Adaptive immune imbalance: Microbiota-derived kynurenine activates the aryl hydrocarbon receptor (AhR), promoting the differentiation of Th17 cells and secretion of IL-17, leading to a Th17/Treg imbalance and increased plaque instability (73). Short-chain fatty butyrate exerts anti-inflammatory effects by inhibiting histone deacetylases (HDACs), enhancing H3K27 acetylation at the *Foxp3* locus, and promoting Treg cell differentiation (74).

### 4.4 Regulation through interorgan signaling axes

(1)    Gut-immune-vascular axis: The gut microbiota-derived metabolite succinate activates hypoxia-inducible factor-1α (HIF-1α) in hematopoietic stem cells within the bone marrow, promoting the differentiation of Ly6C^+^ monocytes. These monocytes infiltrate atherosclerotic plaques and differentiate into pro-inflammatory M1 macrophages (75, 76).
(2)    Gut-neural-cardiac axis: Dysbiosis of the gut microbiota leads to reduced vagal tone and sympathetic nerve activation, resulting in decreased heart rate variability and an elevated risk of myocardial ischemia (77).

The “gut-heart axis” concept has evolved from a single metabolite association of “microbiota-metabolite-cardiovascular injury” framework to a more complex multidimensional network model of “metabolism-immunity-nerve-vascular,” which reveals the importance of gut microbiota as a interventional target in CHD prevention and therapy. Currently, most studies exploring the relationship between the gut microbiota and host health rely on cross-sectional data, lacking prospective longitudinal analyses across multiple time points. Future research should focus on elucidating the long-term relationship between the dynamic changes of gut microbiota and host health and validating the clinical efficacy of the “gut-heart-axis” through randomized controlled trials and optimize the composition of gut microbiota through precision nutritional intervention and use synthetic biology and artificial intelligence to promote the implementation of human microbiome research within the framework of precision medicine, thereby improving host health.

This figure illustrates the complex ecosystem of the gut microbiota and the multiple factors that influence its composition. The intestinal flora is shaped by a combination of host factors (such as genetics, age, and physiological state), dietary factors (including dietary structure, protein sources, and intake of fermented foods and probiotics), environmental factors (like mode of birth, antibiotic use, and geographical conditions), drug exposures (e.g., PPIs, metformin, SGLT2 inhibitors, and immunosuppressants), and lifestyle (such as exercise, stress, sleep, smoking, and drinking). The diagram also highlights key cellular components within the gut mucosa—including dendritic cells, macrophages, and various epithelial cells (e.g., goblet cells, Paneth cells, stem cells)—as well as microbial metabolites and gut hormones, which collectively participate in host-microbe interactions and maintain intestinal homeostasis.

This schematic illustrates the proposed mechanistic pathways through which the gut microbiota influences cardiovascular health. The key pathways involved include:

The Short-Chain Fatty Acid (SCFA) Pathway: Gut microbiota ferment dietary fiber to produce SCFAs. These SCFAs serve as an energy source (ATP) for colonic epithelial cells, enter the portal circulation, and a small fraction reaches the systemic circulation, exerting pleiotropic systemic effects.

The Trimethylamine N-Oxide (TMAO) Pathway: Dietary nutrients are metabolized by gut microbes into trimethylamine (TMA), which is subsequently oxidized in the liver by flavin-containing monooxygenase (FMO) to form trimethylamine N-oxide (TMAO), a pro-atherogenic metabolite.

Immune and Inflammatory Signaling: Microbial-derived pathogen-associated molecular patterns (PAMPs), such as lipopolysaccharide (LPS), can activate pattern recognition receptors (e.g., Toll-like receptors (TLRs), NOD-like receptors). This triggers downstream signaling cascades, including NF-κB and NLRP3 inflammasome activation, leading to the production and release of pro-inflammatory cytokines (e.g., IL-1β, IL-18, TNF-α), thereby driving systemic inflammation that impacts the cardiovascular system.

Nuclear Receptor and Lipid Metabolism: Pathways involving nuclear receptors like the Farnesoid X Receptor (FXR) and key proteins in cholesterol reverse transport such as ABCA1 are also implicated.

The figure integrates these pathways, depicting the complex interplay within the gut-heart axis regulatory network.

## 5 Current perspectives on nutritional management in coronary heart disease

Globally, the rising prevalence of obesity and cardiovascular disease has prompted people to seek for novel and effective dietary strategies. Nutritional management serves as a key component in both primary and secondary prevention of CHD, with its scientific rigor and rational implementation are of great significance in improving lipid profiles, controlling body weight, lowering blood pressure, and reducing the risk of cardiovascular events (14). Elderly patients with cardiovascular disease often face multiple nutritional risks, including metabolic disorders, micronutrient deficiencies, and disease-related dietary restrictions. Studies have revealed that elderly patients with CHD and frailty generally face problems within six months after hospital discharge, such as limited awareness of healthy diet (e.g., entrenched unhealthy dietary habits, passive attitudes toward nutritional management), objective constraints (e.g., taste degeneration, masticatory dysfunction, reliance on subjective appetite to regulate intake, monotonous diets due to living alone), memory impairments, poor self-management capabilities, and discontinuity in professional nutritional guidance (78). In addition, inadequate intake of micronutrients-such as vitamins A, B, C, and E, as well as calcium, magnesium, and zinc may further exacerbate the risk of cognitive dysfunction, decreased immune function and cardiovascular events in elderly patients.

The European Society of Cardiology (ESC) released the Latest Clinical Practice Guidelines for Cardiovascular Disease Prevention in 2021 (79), recommending a diet for CHD patients characterized by low salt intake (< 5 g/day), low fat (saturated fat < 7% of total energy), and high-fiber (> 30 g/day). However, clinical implementation faces multiple challenges: (1) Neglect of Individualized Differences: The metabolic phenotypes of CHD patients are significantly influenced by genetics, gut microbiota composition, and epigenetic regulation, and the traditional “one-size-fits-all” dietary recommendations with broad content and poor precision are difficult to match individual needs. (2) Adherence Challenges: Although an increasing number of healthcare professionals are paying attention to the nutritional status of elderly CHD patients and frail individuals, encouraging adherence to low-salt and low-fat dietary patterns, 68.5% of elderly CHD patients are unable to maintain standard dietary regimens long-term. The major reasons include monotonous recipes, economic constraints and conflicts in cultural dietary habits (80). (3) Lack of dynamic monitoring: Current nutritional assessments primarily rely on static questionnaires, such as food frequency questionnaires (FFQ), which depend on individual memory and self-reporting and are easily affected by recall bias and subjective errors. They lack integration with clinical monitoring indicators and real-time metabolic markers, resulting in delayed intervention and lack of targeting (81).

Nutritional management is a long-term process that requires the concerted efforts of patients, their families, and healthcare teams. Emerging intervention strategies for patients with CHD include targeted modulation of the gut microbiota, metabolomics-driven dietary interventions, the application of digital health technologies, and interdisciplinary collaboration, suggesting that future treatment and nutritional management need to be more precise, combined with lifestyle changes and standardized medical therapies, in order to significantly improve the prognosis and quality of life of CHD patients.

## 6 Bidirectional interactions between gut microbiota and coronary heart disease patients

The gut microbiota and the host exert mutual influences on each other. Certain exposure factors may cause gut microbiota dysbiosis, characterized by a reduction in some intestinal probiotics, ultimately contributing to the development of one or more diseases. On the other hand, during the natural aging process of the body, factors such as declining immune function, alterations in the number and activity of related cells in the intestine will also cause changes in the abundance of gut microbiota. These microbial shifts, in turn, may affect other organs or physiological systems through multiple mechanisms. Given the bidirectional regulatory relationship between gut microbiota and CHD, various dietary interventions have demonstrated cardiovascular protective effects (82–86). We have collated the effects of different dietary patterns on gut microbiota and cardiovascular health (Table 1).

**TABLE 1:** Effects of different dietary patterns on gut microbiota and cardiovascular health.

Dietary pattern	Core characteristics/definition	Impact on gut microbiota	Impact on host metabolism and cardiovascular health
Calorie restriction (CR)	Restriction of total energy intake	•Increases microbial diversity •Enriches beneficial bacteria (100) (e.g., Bifidobacterium, Akkermansia muciniphila) (102) •Reduces abundance of harmful bacteria (e.g., Clostridioides difficile) (101) •Decreases Firmicutes/Bacteroidetes ratio •Reduces TMAO production	•Improves insulin sensitivity •Reduces inflammation •Reduces toxin-mediated metabolic disorders •Closely associated with lipid metabolism
High-fiber diet (HFD)	Rich in complex polysaccharides (dietary fiber), which cannot be directly digested by humans	•Increases microbiota α-diversity (effect varies by fiber type and individual differences) •Enriches SCFA-producing bacteria (e.g., Prevotella, Roseburia, Bifidobacterium) •Reduces conditional pathogens (e.g., Escherichia coli, Desulfovibrio) •Lowers gut pH, inhibiting growth of harmful bacteria •Differential effects of soluble vs. insoluble fiber	•SCFAs (acetate, propionate, butyrate): provide energy for intestinal epithelial cells; regulate immunity and metabolism via GPR41/GPR43 activation and HDAC inhibition (103, 104) •Enhances gut barrier function, reduces LPS translocation, alleviates systemic inflammation (105) •High hPDI shows positive effects in preventing coronary heart disease
Mediterranean diet (MD)	Plant-based diet (high in vegetables, fruits, whole grains, legumes, nuts, olive oil), moderate fish and low-fat dairy, limited red and processed meats	•Significantly increases microbiota α-diversity •Increases Prevotella/Bifidobacterium ratio •Decreases Firmicutes/Bacteroidetes ratio •Exhibits a dose-response relationship •“Green Mediterranean Diet” further optimizes microbial composition	•Mediated via the gut-brain/liver axis: improves insulin sensitivity and provides cardiovascular protection •SCFAs (e.g., butyrate) regulate glucose and lipid metabolism, lower LDL cholesterol and fasting blood glucose •Significantly improves body weight and cardiovascular markers, reducing coronary heart disease risk (83)
Fermented foods and probiotics diet	Intake of fermented foods (e.g., yogurt, kimchi, kombucha) or specific probiotics	•Improves microbial structure: increases α-diversity, inhibits pro-inflammatory bacteria (e.g., Enterobacteriaceae) •Introduces exogenous beneficial bacteria (e.g., Lactobacillus, Bifidobacterium), which may colonize temporarily or long-term (84) •Reduces LPS translocation	•Metabolite regulation: SCFAs (e.g., butyrate) activate GPR41/43, inhibit NF-κB pathway, and improve endothelial function; reduce TMAO levels •Immune regulation: promotes Treg cell differentiation, inhibits Th17-mediated vascular inflammation •Clinical studies show daily yogurt intake reduces cardiovascular disease risk (85, 86)

As human dietary habits result from a specific combination of micro- and macronutrient quantities that are continuously and indefinitely delivered to our gut ecosystem. Assessment and modification of dietary habits can reshape the gut microbiota composition, which in turn influences the host’s mucosal barrier integrity and immune responses. Existing evidence supports a role for gut microbiota regulation in the prevention and treatment of CHD, but most studies are observational in nature. More randomized controlled trials (RCTs) are needed to verify causal relationships. Besides, there are large differences in microbiota between individual, so it is necessary to develop more precise microbiota detection and monitoring technologies, as well as personalized nutritional intervention strategies, to identify optimal dietary patterns, promote a healthy microbiota composition, and maintain host intestinal barrier function and immune function. Although nutrition-based, microbiota-targeted intervention strategies—such as prebiotics, specific dietary fibers, or functional foods—exhibit considerable application potential, translating promising hypotheses into universally applicable clinical practices hinges on advancing methodological standardization and rigorous clinical validation. This necessitates, first and foremost, the establishment of fully standardized microbiota detection protocols encompassing sample collection, preservation, DNA extraction, and bioinformatic analysis to minimize technical variability and its impact on results. Building upon this foundation, it is imperative to conduct large-scale, well-designed randomized controlled trials in clearly defined populations to systematically evaluate the causal effects of these nutritional interventions on specific microbial community structures, functions, and associated host health indicators. Only through such a rigorous scientific pathway can we obtain compelling, high-quality evidence to accurately validate the true benefits of these microbiota-targeted nutritional strategies and ultimately facilitate their effective application in precision nutrition and chronic disease management.

## 7 Nutritional supports for patients with coronary heart disease has shifted toward a multi-target, integrated intervention model

Based on evidence-based medicine and precision nutrition strategies, nutritional management for patients with CHD has shifted from traditional single-nutrient control to a multi-target, individualized comprehensive intervention model. The core advancements are reflected in breakthrough developments across the following four key dimensions:

### 7.1 Metabolomics-driven precision nutritional modulation

#### 7.1.1 Targeted remodeling of the gut microbiota

The gut microbiota directly regulates immune cell function through its metabolites, such as short chain fatty acids (SCFAs) and indole derivatives. Strategies such as probiotics, prebiotics, and fecal microbiota transplantation (FMT) have been used to restore gut microbiota balance, modulate inflammatory responses and immune tolerance. Modulating the gut microbiota through dietary interventions (such as high-fiber diet or fermented foods) has also been shown to affect immune status. A study based on NHANES data demonstrated that for each 1-point increase in the Dietary Inflammatory-Gut Microbiota (DI-GM) index score, the risk of CHD decreased by 4% (87). The key molecule of ginsenoside Rb1 has been shown to regulate the gut microbiota and mitochondrial function via the DUSP-1-TMBIM-6-VDAC1 axis, suppress the release of pro-inflammatory cytokines, and protect damaged heart function (88).

#### 7.1.2 Precise inhibition of pathogenic metabolites

Thylamine N-oxide-targeted modulation: Nutritional strategies that inhibit gut microbiota trimethylamine (TMA) production—such as supplementation with 3,3-dimethyl-1-butanol and low-choline/carnitine diets—can significantly reduce plasma TMAO levels (by 42%) and reverse coronary plaque inflammatory activity, resulting in a 1.8-fold increase in coronary artery plaque stability (89).

Dynamic monitoring of branched-chain amino acids (BCAAs): By combining continuous glucose monitoring (CGM) with serum metabolomics analysis and AI-based algorithms to optimize protein sources (e.g., replacing red meat with plant-based proteins), the BCAA/keto-isocaproate ratio serves as an important indicator of BCAA metabolic status. Dietary structure and protein source adjustments can lower the BCAA/keto-isocaproate ratio and improve insulin sensitivity (90).

### 7.2 Innovation in evidence-based dietary pattern implementation

#### 7.2.1 Region-specific dietary adaptations

Multiple studies have confirmed that the Mediterranean dietary pattern has significant protective effects on cardiovascular health. A large 7-year randomized controlled trial (CORDIOPREV) demonstrated that, compared to the low-fat diet group, the Mediterranean diet group had a 25%–28% reduction in the risk of cardiovascular events, with particularly significant effects in male (91). Long-term (5–7 years) adherence to the Mediterranean diet can significantly reduce carotid intima media thickness (IMT-CC) and slow the progression of atherosclerosis. However, the Mediterranean diet is not applicable globally, especially dietary habits among elderly individuals in Asia (92, 93), and there is an urgent need to seek intervention models that favor the long-term nutritional management of patients with CHD.

#### 7.2.2 Clinical translation of time-restricted eating (TRE)

In addition to limiting the types and cooking methods of healthy diets for CHD patients, regular meal timing, moderate portion sizes, safe cooking practices, and a pleasant dining environment all contribute to reducing the incidence of CVD. Maintaining regular meals, especially consistently eating breakfast, can reduce the risk of CHD by 27% (94), whereas eating within 2 h before sleep or during late night hours may increase CHD risk by 55%. BMAL1 and CLOCK are core circadian rhythm genes that play a key roles in maintaining metabolic and cardiovascular health. In patients with coronary heart disease (CHD), the upregulation of the BMAL1/CLOCK gene may promte the restoration of vascular relaxation function by restoring circadian rhythms in endothelial cells. Time-restricted feeding (TRF), such as a 10-h eating window interventions (e.g., 8:00–18:00), can increase the improvement rate of endothelial function in CHD patients by 2.3 times through the regulation of circadian rhythm genes (BMAL/CLOCK) (95).

### 7.3 Nutrition-drug synergistic enhancement system

Lipid-lowering synergy: Oat β-glucan is a soluble dietary fiber that can affect cholesterol metabolism by modulate the gut microbiota and increase the production of short-chain fatty acids (SCFAs), thereby affecting cholesterol metabolism. A daily intake of 10 g oat β-glucan has been shown to enhance the LDL-C reduction effect of atorvastatin and activate hepatic LDL receptor expression, contributing to better lipid control (96).

Metabolic remodeling co-intervention: SGLT2 inhibitors (SGLT2i) lower blood glucose levels by inhibiting renal glucose reabsorption and promoting urinary glucose excretion (97). A low-carbohydrate diet reduces glycaemic fluctuations and improves insulin sensitivity by restricting carbohydrate intake. The combination of these two approaches not only significantly improves metabolic parameters but also optimizes cardiac energy metabolism, thereby reducing the risk of heart failure. This effect is associated with ketone metabolism remodeling and enhanced myocardial energy efficiency.

### 7.4 Digital health technologies empowering precision management

Dynamic monitoring via wearable devices: In addition to tracking routine vital signs, smart wearable devices can collect data on dietary intake, physical activity, and weight changes. Clinicians and nutritionists can remotely access these data along with clinical indicators such as blood lipids, blood pressure, and glucose levels to assess overall risk management effectiveness. AI-based platforms integrating continuous glucose monitoring (CGM), heart rate variability (HRV), and gut microbiota testing analysis (such as Cardio-NutriNet) can predict individual dietary responses and guide dynamic adjustments to nutritional plans (98).

Management systems based on the ChatGPT-4 architecture can identify deficiencies in 12 micronutrients in real time. Based on a patient’s health status, dietary habits, and nutritional needs, the system generates personalized dietary plans. By analyzing daily intake data, it can detect specific micronutrient deficiencies and recommend targeted foods or supplements. This dynamic adjustment mechanism, supported by telemedicine collaboration, ensures that patients receive continuous and effective nutritional support, thereby improving long-term dietary adherence (99).

## 8 Conclusion and prospect

Metabolites produced through microbial fermentation of nutrients can directly or indirectly influence the composition and metabolic activity of the gut microbiota in CHD, thereby exerting profound effects on their overall health. Based on the “gut-heart axis” theory, nutritional intervention has emerged as a potential strategy for modulating the gut microbiota and improving CHD prognosis. Currently, nutritional support has entered a precision era characterized by “dynamic monitoring-algorithm decision- targeted intervention.” However, its implementation still faces several challenges: (1) The majority of CHD patients are elderly, with entrenched dietary habits and low adherence to post-illness nutritional guidance. (2) There are insufficient interdisciplinary collaboration-clinical physicians often lack effective communication with dietitians, making it difficult to develop precise, individualized nutritional plans. (3) Standardization of microbiota testing techniques is lacking, and there is a shortage of long-team efficacy data from intervention studies. (4) Precision medical testing remain costly, with limited coverage under current healthcare reimbursement systems.

With the rapid advancement of genomic sequencing and artificial intelligence technologies, it is now feasible to establish a CHD-specific gut microbiota biomarker profile for CHD patients and to develop personalized dietary prediction models based on machine learning algorithms. By integrating multi-omics data including metagenomics, metabolomics, and clinical indicators, researchers can develop portable rapid microbiota detection tools, such as microfluidic chip technologies, constructing a three-pronged management model of “cardiology-nutrition-digital platform,” along with the exploration of tiered healthcare payment schemes. With continued progress in single cell sequencing and AI technology, future innovations may include wearable devices capable of monitoring microbial metabolic status and dynamically adjusting the diet, as well as the use of engineered probiotic strains (e.g., butyrate-producing gene-edited bacteria) to precisely correct microbial imbalances. Furthermore, the establishment of regional CHD nutritional intervention databases may guide public health policies and enhance health literacy level among CHD patients.
