# Correction: Advances in nutritional interventions for coronary heart disease patients from the perspective of the gut-heart axis

**DOI:** 10.3389/fnut.2026.1779910

**Published:** 2026-01-28

**Authors:** Jing Gao, Mingjing Zhang, Gaoning Zhang, Dingzheng Zhang, Mo Zhou, Lijing Zhao, Yanwei Du

**Affiliations:** 1 School of Nursing, Jilin University, Changchun, Jilin, China; 2 Department of Nursing, First Affiliated Hospital of Xinjiang Medical University, Urumqi, Xinjiang, China; 3 Institute of Orthopaedic & Musculoskeletal Science, Royal National Orthopaedic Hospital, University College London, London, United Kingdom

**Keywords:** gut-heart axis, coronary heart disease, nursing, nutrition, gut microbiota

Author Mingjing Zhang's designation as a co-first author was erroneously omitted.

The original version of this article has been updated.

